# The effectiveness of a smart school bag system for reminding students of forgotten items and reducing the weight of their bags

**DOI:** 10.1186/2193-1801-4-S2-O2

**Published:** 2015-11-27

**Authors:** Sau-ming Lau, Yammy Wai-yan Wong, Fiona Wing-yin Luk, Stella Sin-tung Kwok

**Affiliations:** Research Support Unit, Vocational Training Council, Hong Kong

## Background

A learning support system integrated in a school bag was developed to help primary school students manage their personal items. The smart school bag is an illustration of the application of information and computer technology. Using tailor-made sensors and a tablet embedded in students' school bags, users (including teachers, parents and students) could maintain a daily schedule for a list of school items. Using near field communication, each item inside the bag was identified and recorded. The tablet then compared the list of items with the objects in the bag and provided a reminder for the student when an item was missing. This presentation introduces the smart school bag system and illustrates the effectiveness of the system with the use of a mixed methods experiment.

## Methods

Semi-structured individual interviews were conducted with 50 primary school students aged 6-8 years old from 1-9 November 2014 to explore their experiences (e.g. how often they forgot things in daily life, the effectiveness of the school bag and how much they thought it had modified their behaviour). Based on the results of this exploratory study, the authors tested the accuracy and usability of the system with the use of an experiment. The experiment involved 30 students who were invited to test the school bag system by using it together with their guardians. After the experiment, the pupils and their guardians filled out the same multiple-choice questionnaire separately. The interview transcripts were analysed using NVivo 9 and the questionnaire data were analysed using SPSS.

## Results

The interview findings revealed that the participants were amazed that the smart school bag could actually speak and provide interactive responses and reminders to users. They thought that the smart school bag was convenient to use but rather expensive because it also required a tablet. The users suggested that a reminder service could be provided for other objects apart from books, including water bottles, lunch boxes and sportswear. According to the results of the quantitative questionnaire, all of the students and 90% of their guardians believed that the smart school bag helped them to avoid forgetting the objects they needed to take to school. All of the students and guardians agreed that the smart bag helped the students to get into the habit of tidying up their school bags and reduced the weight of them. More than 30% of the guardians worried that the use of smart school bag might reduce students' personal management ability if they relied too much on the technology.

## Conclusions

The results of the experiment demonstrate that the smart school bag has potential for use in real life applications. It could also be used to collect data about students' daily behaviour. In future studies, the principles of behavioural psychology and vertebral ergonomics could be integrated into the system to further enhance the smart school bag.

